# Robust Linear Models for Cis-eQTL Analysis

**DOI:** 10.1371/journal.pone.0127882

**Published:** 2015-05-18

**Authors:** Mattias Rantalainen, Cecilia M. Lindgren, Christopher C. Holmes

**Affiliations:** 1 Department of Statistics, University of Oxford, Oxford, United Kingdom; 2 Wellcome Trust Centre for Human Genetics, University of Oxford, Oxford, United Kingdom; 3 MRC Mammalian Genetics Unit, MRC Harwell, Harwell, United Kingdom; Queen’s University Belfast, UNITED KINGDOM

## Abstract

Expression Quantitative Trait Loci (eQTL) analysis enables characterisation of functional genetic variation influencing expression levels of individual genes. In outbread populations, including humans, eQTLs are commonly analysed using the conventional linear model, adjusting for relevant covariates, assuming an allelic dosage model and a Gaussian error term. However, gene expression data generally have noise that induces heavy-tailed errors relative to the Gaussian distribution and often include atypical observations, or outliers. Such departures from modelling assumptions can lead to an increased rate of type II errors (false negatives), and to some extent also type I errors (false positives). Careful model checking can reduce the risk of type-I errors but often not type II errors, since it is generally too time-consuming to carefully check all models with a non-significant effect in large-scale and genome-wide studies. Here we propose the application of a robust linear model for eQTL analysis to reduce adverse effects of deviations from the assumption of Gaussian residuals. We present results from a simulation study as well as results from the analysis of real eQTL data sets. Our findings suggest that in many situations robust models have the potential to provide more reliable eQTL results compared to conventional linear models, particularly in respect to reducing type II errors due to non-Gaussian noise. Post-genomic data, such as that generated in genome-wide eQTL studies, are often noisy and frequently contain atypical observations. Robust statistical models have the potential to provide more reliable results and increased statistical power under non-Gaussian conditions. The results presented here suggest that robust models should be considered routinely alongside other commonly used methodologies for eQTL analysis.

## Introduction

Expression Quantitative Trait Loci (eQTL) analysis [1–3] provides important study designs in functional genomics as they enable the characterisation of genetic sequence variants, commonly Single Nucleotide Polymorphisms (SNPs), that associate with mRNA expression levels of individual genes. Determining if mRNA expression levels are driven by specific genetic variants provides evidence of a functional and mechanistic link between genetics and downstream molecular events, of which the first step is changes in gene expression. EQTLs therefore have the potential to provide functional interpretation of SNPs that are associated with a phenotypic trait, but also to provide more general information about how genetic variation influences gene expression. EQTL analysis has been applied in a number of organisms, including human [4, 5], mice [6, 7] and rats [8, 9], and has revealed that a substantial proportion of mRNA expression levels are influenced by genetic variation. EQTL analysis is carried out in either inbread populations, such as laboratory mice, or outbread populations, such as humans. In this paper we focus on the case of outbread populations, which include humans and is therefore of particular interest in biomedical applications. EQTL analysis is commonly pursued using a linear model based on the allelic dosage model assuming an additive genetic effect as a function of the number of effect alleles [3, 10–15]. The main objective in such analyses is to test for evidence against the null hypothesis *H*_0_:*β*_*SNP*_ = 0, where *β*_*SNP*_ encodes the regression coefficient associated with the genetic variant, in order to ascertain if there is a significant genetic effect on the mRNA expression level. It is often relevant to adjust the model for known covariates, for example, gender, age, body mass index, disease status and batch effects [10], particularly in epidemiological and human studies. This can easily be performed in a linear model. Common among all genome-wide eQTL studies, including those only focusing on cis-effects, is that a high number of genetic variants, and hence models, are evaluated, ranging from hundreds of thousands to several millions. Large-scale analyses effectively prohibit careful manual checking of each model, which is otherwise advisable to avoid severe deviations from assumptions. Manual model checking [16, 17] procedures, often based on visual inspection, commonly include detection of extreme or outlying observations, high leverage points and deviations from assumed distributional assumptions. Deviations from underlying model assumptions can lead to both type I and type II errors. Type I errors (false positives) can occur due to biased genetic effect size estimates due to extreme observations or outliers either in gene expression data or in the genetic data. Type II errors (false negatives) can arise in the same way, due to biased effect size estimates, or more commonly, due to inflated estimates of the standard-error. Here we focus on assessing the effectiveness of robust alternatives to the conventional linear model for genome-wide *cis*-eQTL analysis. We consider a robust alternative to the linear model that is based on the MM-estimator [18, 19] and compare results with the conventional linear model. Results are first presented from a simulation study where we evaluate statistical power of the two models in data simulated from a contamination model as well as a heavy-tailed model. Finally we present results from the comparative study between the standard and the robust model based on two real eQTL data sets.

## Materials and Methods

### Linear model

The conventional linear model (Eq 1) with a Gaussian error term is widely used for association analysis in biological applications, including eQTL analysis.

$$\begin{array}{c} y=\mu +X\beta +\epsilon \end{array}$$ (1)

Where **y** is a vector of expression values [*N* × 1] for N observations, *μ* is the intercept, **X** is the design matrix of dimension [*N* × *k*] for *k* covariates and *ϵ* is a vector [*N* × 1] containing the error term. **X** encode the SNP effect and any other relevant covariates. The linear model is generally fitted by maximum likelihood, corresponding to the least squares solution (Eq 2), assuming that *ϵ* ∼ *N*(0, *σ* ^2^). We will refer to the least squares model as the “conventional” model throughout this paper.

$$\begin{array}{c} {\hat{\beta }}_{LS}=\underset{\beta }{\mathrm{argmin}}\sum_{i}^{n}{\left(y_{i}-\mu -X_{i,}\beta \right)}^{2} \end{array}$$ (2)

It is well known that the linear model has a breakdown point of 0 [20]. The breakdown point can be understood as the proportion of outlying or extreme observation the estimator can tolerate before giving incorrect and arbitrarily large results. If the breakdown point is 0, then a single extreme outlier can have an unbounded effect on the estimate of ${\hat{\beta }}_{LS}$, suggesting that the linear model may not be suitable when data is likely to contain either outlying observations or when the error term is not necessarily expected to be Gaussian, for example in noisy biological data.

### Robust linear model

Robust models are characterised by being resistant to deviations from the common distributional assumptions, such as that of Gaussianity of the error term in the conventional linear model. Robust models facilitate, sometimes substantial, improvements of inferences in presence of outliers, or other deviations from common model assumptions. In general robust models also maintain relatively high efficiency in the case when there are no deviations from assumptions in the conventional model. In data where the conventional assumptions are unlikely to be met robust alternatives are likely to provide improved results. Biological data sets often contain data that do not necessarily follow e.g. Gaussianity, and robust models can provide more reliable inferences over conventional models in analysis of this type of data.

### MM-estimators

The M-estimator (maximum likelihood-like estimator) is a general class of estimators calculated as the minima of sums of a function (Eq 3).

$$\begin{array}{c} {\hat{\beta }}_{M}=\underset{\beta }{\mathrm{argmin}}\sum_{i}\;\rho \left(x_{i},\beta \right) \end{array}$$ (3)

Where the function *ρ* is chosen to provide properties of robustness. Common examples of *ρ* for robust models is the Huber function [20] and the bisquare function [20], which we use in this study (Eq 4). The bisquare function has the ability to reject gross outliers (effectively given them a weight equal to 0), while more moderate outliers are down weighted smoothly, thus providing properties of robustness and efficiency [20]. The choice of *ρ* is generally based on the expected properties of the data as well as the trade-off we are willing to make between e.g. robustness and efficiency. Provided that gene expression data may have extreme outliers as well as heavy-tailed noise, the bisquare redescending function provides a reasonable choice. The Huber function in contrast will not lead to completely rejecting extreme outliers, instead they are down weighted in the model, and therefore this function can be more sensitive to presence of extreme outliers.

$$\begin{array}{c} \rho \left(x\right)=\{\begin{array}{cc} 1-{\left(1-{\left(x/k\right)}^{2}\right)}^{3} & \text{if}\;|x|\leq k\\ 1 & \text{if}\;|x|>k \end{array} \end{array}$$ (4)

In Eq 4 the parameter *k* is chosen to achieve the desired efficiency, i.e. the precision of the estimate relative its theoretical limit (e.g. 95% under Gaussianity). We note that *k* is not optimised during model fitting.

The M-estimator is robust towards extreme values in **y**, but not resistant to high leverage points in **X** [21]. When **X** is a design matrix, or represent only genetic information **X** ∈ {0, 1, 2}, this is not an issue. However, in the case when **X** is random, outliers may influence the M-estimate. The MM-estimator is an extension to the M-estimator that provides robustness in respect to both outliers and to some extent high leverage points [21]. In eQTL studies, the models may include, in addition to the genetic information, covariates representing phenotypic information, which may have outliers. In addition, we note that genotype calls have some associated uncertainty, further motivating the choice of the MM-estimator [18, 19]. The MM-estimator proceeds by first fitting a robust scale estimate based on an S-estimate [22], and subsequently the robust scale estimate is held constant while an M-estimate for the location is estimated. Numerically estimates are calculated using the iteratively reweighted least squares (IRWLS) method. In the case of eQTL models, both **y** (gene expression) and **X** (genotypes and other covariates) may have outliers or extreme values. For the purpose of the current study we will be employing the MM-estimator, using a bi-square redescending score function (Eq 4), with standard errors calculated as described in [23]. The hypothesis tests and associated p-values from the robust model are based on an asymptotic approximation using robust estimates of the location and scale parameters [23]. The robust model has a breakdown point of 50% and 95% asymptotic efficiency in the case of Gaussian errors. All analyses were carried out using R [24], including functionality provided in the robust package (see S1 Text for example code for fitting robust linear models in R). We will refer to the linear model estimated through the MM-estimator as the “robust model” throughout this paper.

## Results

### Simulation study

**Simulation study 1: contamination model** We evaluated the statistical power, i.e. the probability that the hypothesis test will reject the null hypothesis when the null is false, for the conventional and robust linear model using data simulated from a mixture contamination model (see below). The biological relevance of the mixture contamination model is the assumption that a small proportion of extreme observations, outliers or otherwise atypical observations, are present in the data. Such extreme or atypical observations can arise due to experimental artefacts (a conventional outlier), due to stochastic properties of the biological system (e.g. a natural extreme value) or due to e.g. biological contamination of biopsies so that in some samples a substantial proportion of the biopsy contains a different tissue type than intended.

We assumed an eQTL model with a linear additive genetic effect, *Y* = *Xβ* + *ϵ*. Where *X* is the allele count (0, 1, 2), *β* is the genetic coefficient and *ϵ* are the residuals, which are assumed to be ∼ *N*(0, *σ* ^2^). We simulated contaminated data by drawing observations from the true model with probability *π*, and from a contamination distribution with probability 1 − *π* (here *π* = 0.95 unless otherwise stated). The contamination distribution had variance equal to *κσ* ^2^, with *κ* = 10 and *β* equal to 1, unless otherwise stated. 10,000 simulation rounds were performed to assess power and 1 * 10^6^ simulation rounds performed to assess the type I error rate.

**Fig 1:**
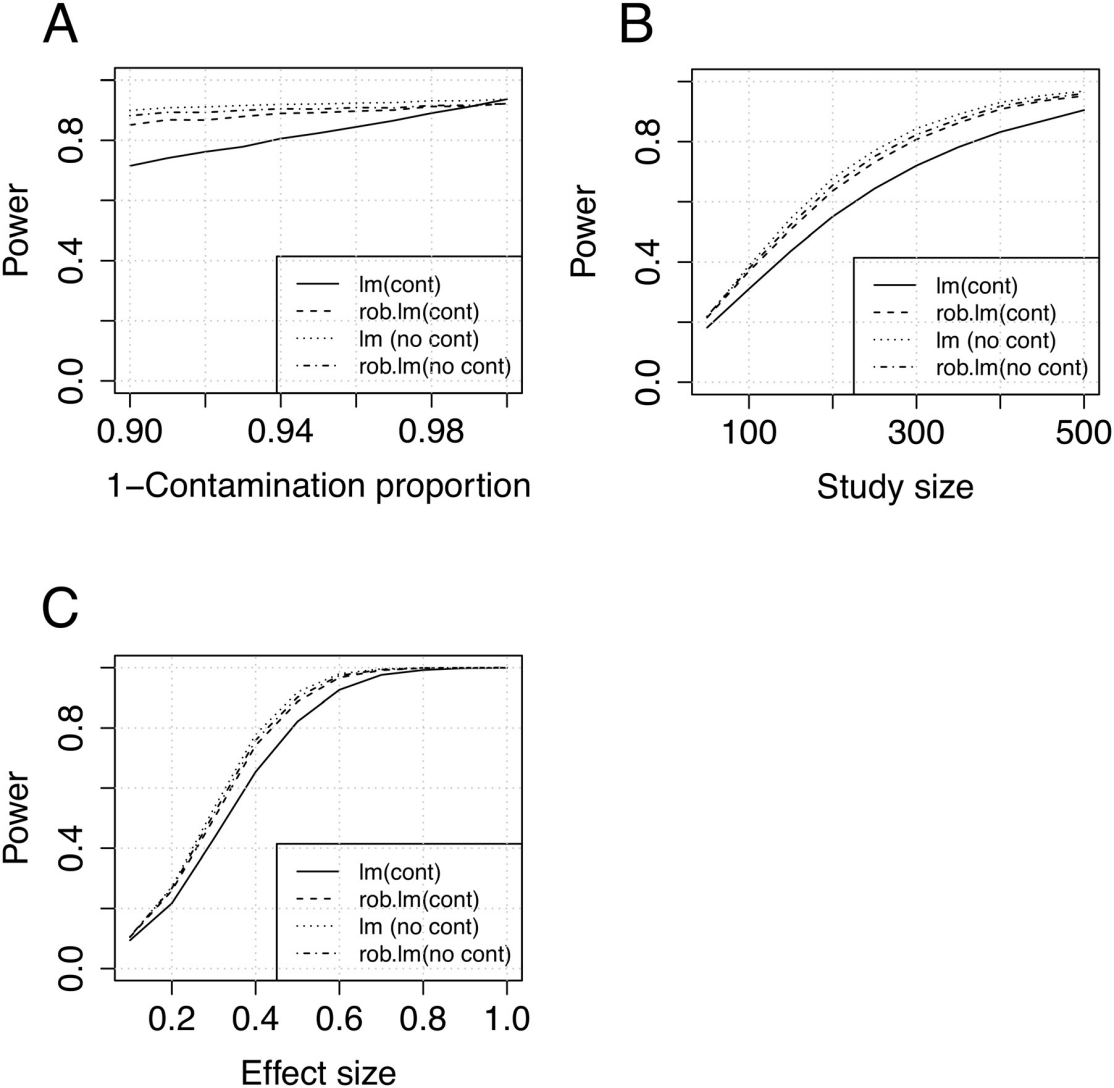
Power analysis results (mixture contamination model). A) Power as a function of contamination proportion. B) Power as a function of study size. C) Power as a function of the genetic effect size. (Simulation parameters: 10000 samples; A, B and D: N = 100; B, C and D:*π* = 0.95)

First, we evaluated the power as a function of the contamination proportion (1 − *π*) in the range 0–0.1. The resulting power curves can be found in Fig 1A), suggesting a relatively high sensitivity of the conventional linear model even to small proportions of contamination. Close to the Gaussian situation (< 1% contamination), the conventional model has slightly higher power than the robust alternative, while the robust model has higher statistical power under higher proportion of contamination. Next we considered the impact of sample size on statistical power for both models (Fig 1B). These results indicate a potential gain in statistical power in the case of contaminated data using the robust model. Lastly, we assessed the power as a function of the genetic effect size (Fig 1C), at fixed effect sizes = {0.1, 0.2, …, 1}, demonstrating a gain in statistical power as a function of the effect size.

From these results we note that the conventional model was relatively sensitive to even small proportions of deviations from the underlying assumptions, here in the form of contamination of the error term, while the robust alternative has power close to the conventional model also under the idealised model assumptions (the MM-estimator applied here has 95% efficiency in the case of Gaussian errors). This means that even if the data do not deviate from the assumptions, there is a relative small loss (Fig 1A, 1B and 1C) in statistical power using the robust model. In respect to power as a function of study size, we found that under these simulations a substantial increase in power (Fig 1A, 1B and 1C) is gained by using the robust model. Under the contamination model we could of course ignore the deviations from the model assumptions, apply the conventional model and then compensate by increasing the study size to achieve the same statistical power. This would, however, lead to unnecessary loss in efficiency and increase in experimental study costs. Both models were confirmed to have false positive rates close to the expected rates (S1 Table), based on 1 * 10^6^ simulation rounds. The mean and standard deviation of effect size estimates from the simulations were evaluated at 5% contamination, indicating slightly larger standard deviation in the case of the conventional model (S2 Table) in the contaminated situation.

**Fig 2:**
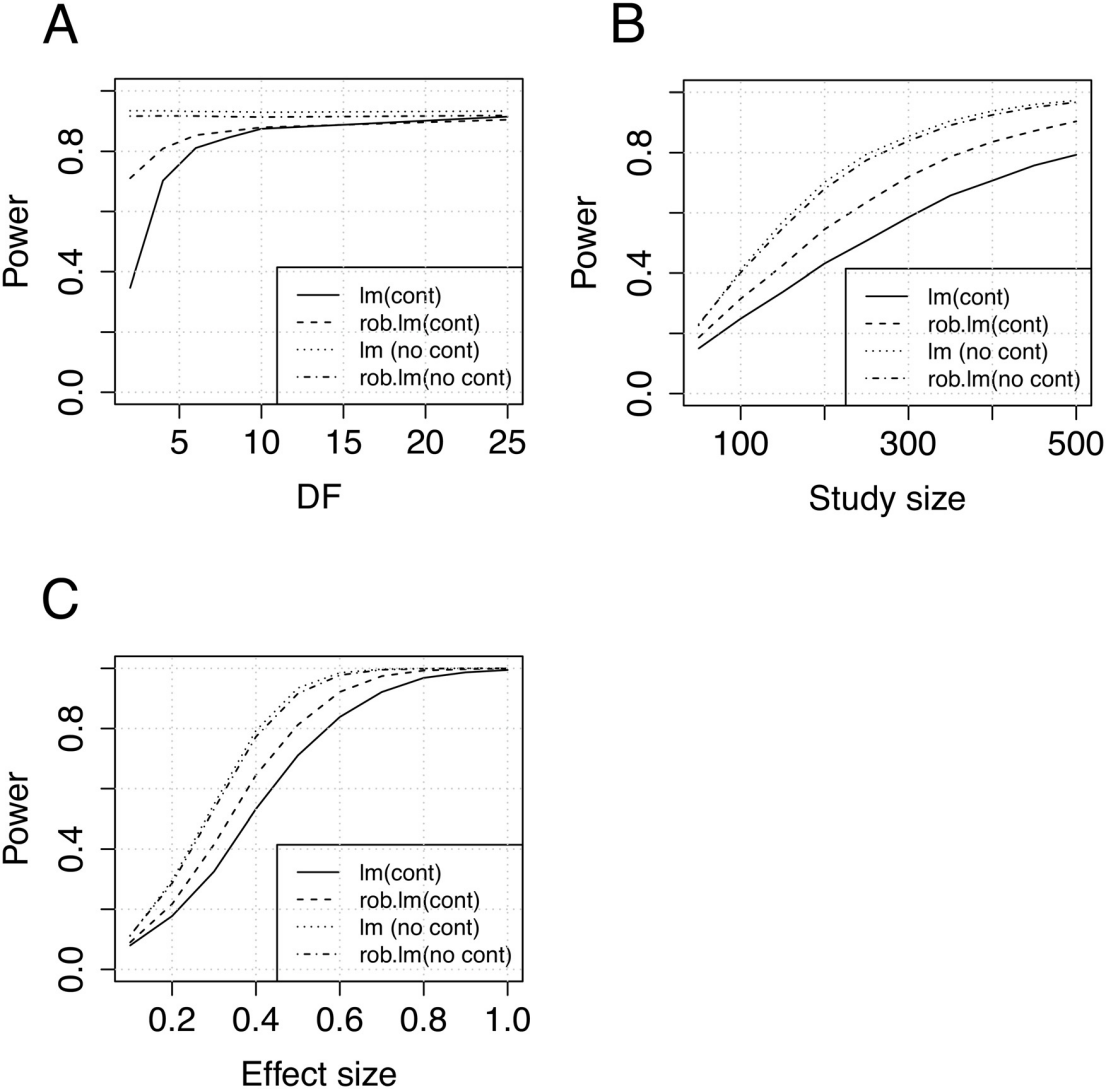
Power analysis results (heavy-tailed). A) Power as a function of degrees of freedom in the student t-distribution. B) Power as a function of study size. C) Power as a function of the genetic effect size. (Simulation parameters: 10000 samples, A-B, D: N = 100, B-D:*df* = 4)

**Simulation study 2: heavy-tailed model** In the second simulation study data were simulated with a heavy-tailed noise term using the student t-distribution with few degrees of freedom. In all other aspects the simulation setup was similar to the mixture contamination model described above. First, we evaluated the statistical power as a function of the degrees of freedom in the t-distribution, in the range 1–25 (Fig 2A). The results indicate how the conventional model start to lose power at df < 15, and in the case of df < 10, when data are starting to be substantially heavy-tailed, the robust model was found to have substantially higher power than the conventional model. Next, we considered the impact of sample size on statistical power for both models (Fig 2B). Our results indicated that statistical power improves with the robust model relative to the conventional model if the noise term is heavy-tailed. Lastly, we assessed the power as a function of the genetic effect size (Fig 2C). Both models were confirmed to have false positive rates close to the expected rates (S3 Table) based on 1 * 10^6^ simulation rounds.

Also under simulations with a heavy-tailed error term, our results indicated better power of the robust model relative to the conventional model, however, the gain in power was somewhat smaller than in the contamination scenario in the previous section. Nevertheless, both simulation setups indicated an increase in statistical power using the robust model under non-Gaussian conditions, while the loss in power was found to be relatively small under Gaussian conditions. This suggests that many real studies with noisy data, e.g. expression data in eQTL studies, are likely to benefit from the application of robust statistical models.

**Fig 3:**
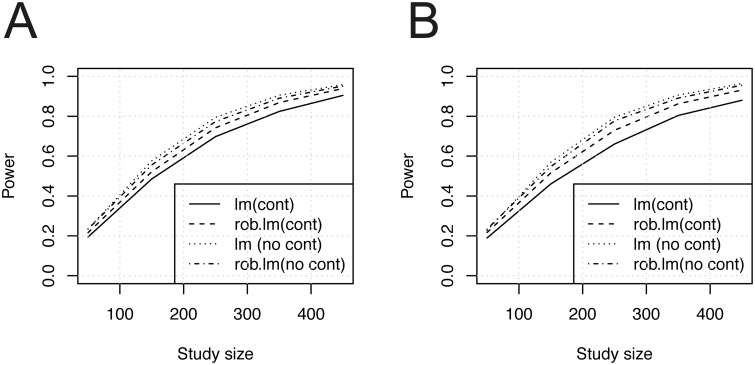
Power analysis results (empirical residuals from robust model fit). A) Residuals from a random sample of eQTL models. B) Residuals from a random sample from models found to be significant only in the robust eQTL model. (‘cont’ = residual from robust model fit of Myers *et al*. [25] data set; ‘no cont’ = Gaussian residuals.)

**Simulation study 3: empirical residuals from a robust model fit of real biological eQTL data** In a third simulation study, utilising empirical residuals from a robust model fit of real eQTL data, we compared power between the conventional and the robust linear model followed the previous simulations. Here the error terms were sampled from robust residuals obtained from a real biological data set [25] (for details about this data set, see next section). For each of the 10,000 simulation rounds, a robust model was fitted to a randomly sampled real data mRNA-SNP eQTL pair. The residuals were extracted and subsequently a random sample with replacement from these residuals were drawn and used as the error term in the data generated in this simulation. The effect size was set to 0.25 throughout this simulation and the residuals from each eQTL model were centred by the median and scaled by the median absolute deviation. Fig 3 shows statistical power as a function of the sample size under these conditions. The results suggest that the error term used in this simulation do to some extent deviate from the Gaussian case, also included in the simulation (Fig 3). We observed that the robust model consistently had better power than the conventional model when we sampled the residuals from the real eQTL data set. We also found that the discrepancy in power between the models were greater in the case of sampling from the subset of eQTL models that were found to be significant only by the robust model (3B).

### Comparative analysis of real biological eQTL data

Following on the simulation studies we analysed two real biological eQTL data sets. Our focus was to assess the concordance between eQTL results from the conventional linear model and the robust model in analysis of real biological eQTL data set in addition to the above reported simulations.

### Real biological eQTL data set 1: Myers *et al*

The first data set is publicly available and was published by Myers *et al*. [25], who applied a conventional linear model for analysis. The study contains data from expression measurements from 193 neuropathologically normal human brain tissues, see [25] for details.

**Fig 4:**
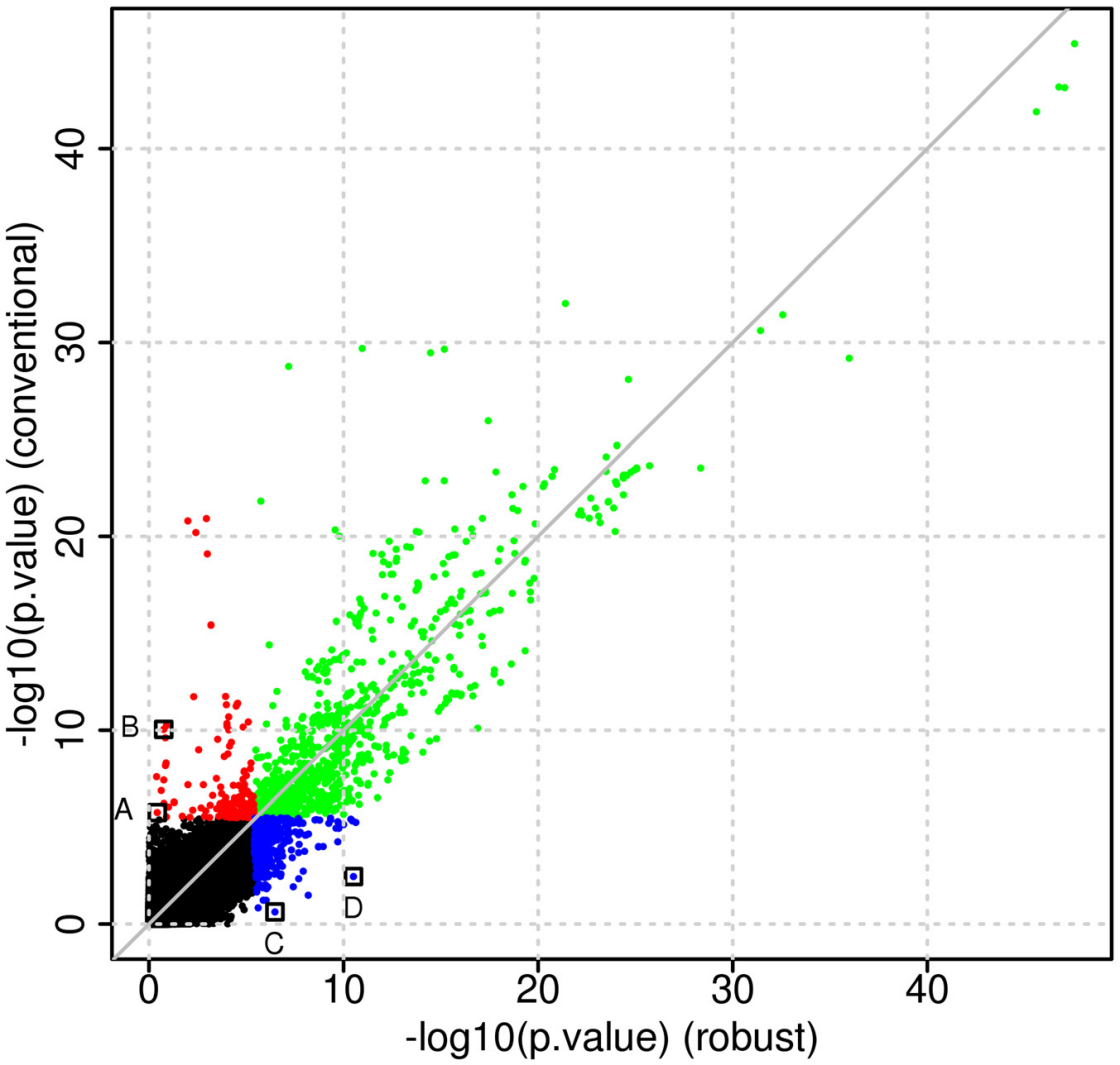
P-value correspondence in Myers *et al*. data set [25]. Scatter plot of −*log*_10_(p-values) from Myers *et al*. data set [25]. (Key: green = significant in both models, red = significant in the conventional model only, blue = significant in the robust model only, data from points marked with black squares are shown in Fig 5)

**Fig 5:**
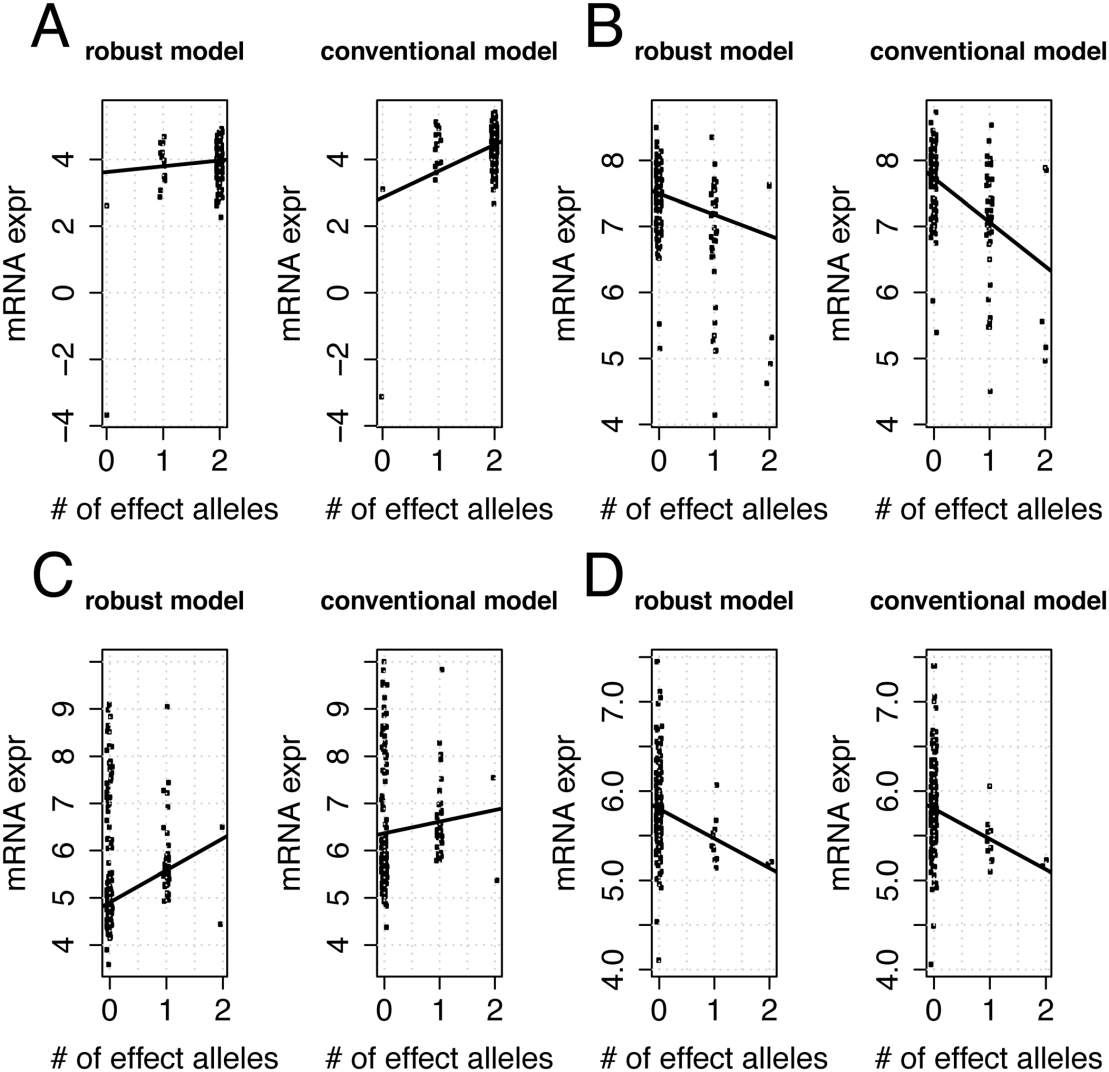
Examples of discordant results in Myers *et al*. data set [25]. Discordant eQTL calls by the conventional and robust model in the Myers *et al*. data set. Labels A-C correspond to marked points in Fig 4. (Note that a small amount of random variability (jitter) has been added in the x-axis direction to better visualise data. MRNA expression levels (y-axis) represent $y_{i}-x_{i}{\hat{\beta }}_{covariates}$, where ${\hat{\beta }}_{covariates}$ are the estimated coefficients for all predictors in the model excluding the genetic effect, i.e. the mRNA signal after adjusting for covariates. Since the conventional and robust estimates of ${\hat{\beta }}_{covariates}$ will be different, the data points in the plots for the robust and conventional models will not be identical.

We performed a genome-wide eQTL analysis of cis-eQTLs (+/- 1Mb window relative the transcription starting site of each gene) using both the conventional linear model and the robust model. Both models were adjusted for the following covariates: gender, age at death and brain region where the tissue samples were taken. To study concordance between the results from the two models, we selected those eQTLs where at least one of the two models indicated a significant genetic effect (FDR adjusted p-value < 0.01). We stratified this set of eQTLs into three groups: significant SNP effect in both models, significant SNP effect in the conventional model only and significant SNP effect in the robust model only (Fig 4). The number of models falling into either of these groups are listed in Table 1. The numbers represent mRNAs with at least one associated SNP, which does not have to be the same in the conventional and the robust linear model. These results indicate that only 50% of the eQTL genes are common between the conventional and robust model, a relatively low level of concordance. To provide further illustration of situations where the conventional linear model and the robust model lead to discordant conclusions regarding significance of the genetic effect, we plot results from four particular eQTLs (Fig 5). The corresponding points are also marked in Fig 4. Fig 5A and 5B show examples of inflated absolute genetic effects due to one or a few extreme observations, while Fig 5C shows an example of reduced absolute genetic effects due to extreme observations. Fig 5D illustrates an example with similar genetic effect sizes between the two models, while the standard error is inflated in the conventional model due to presence of tail events leading to a non-significant test.

**Table 1:** Concordance (number and proportion of mRNAs with at least one eQTL SNP) between the conventional and robust models (Myers et al. data set [25].

	Both	L.M. unique	Rob. unique
Proportion eQTLs	0.50	0.12	0.38
Number eQTLs	145	35	112

Key:both = significant in both models, L.M. unique = significant in conventional model only, Rob. unique = significant in robust model only).

**Fig 6:**
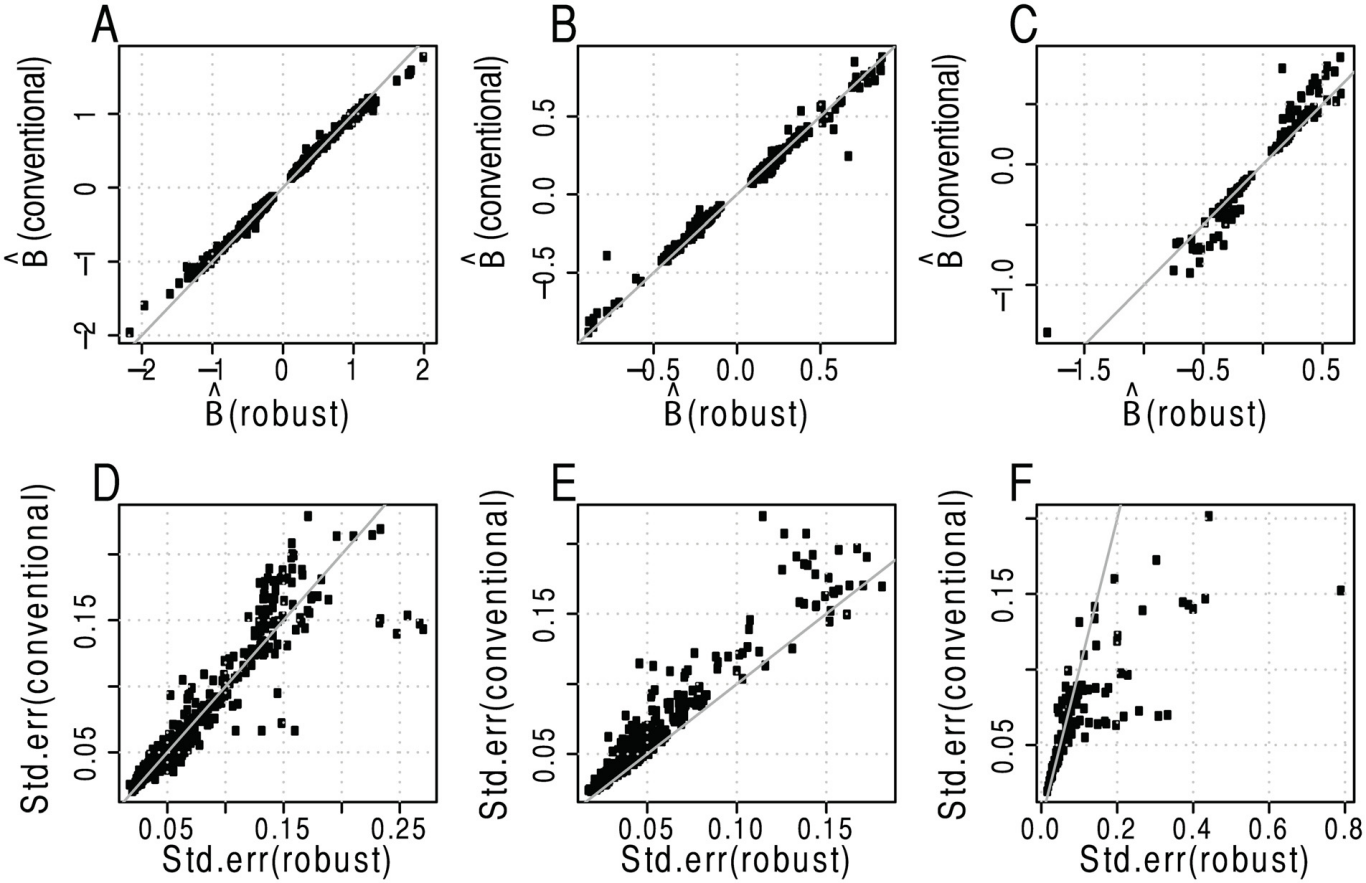
Results from comparative analysis of Myers *et al*. data set [25]. SNP effect size estimates and standard errors for eQTLs significant in both models (A, D), in the robust model only (B, E), and in the linear model only (C, F).

We then proceeded to further determine the nature of the discordance between the conventional and the robust model, in particular we inspected the genetic effect size estimates and related standard error estimates from the conventional and robust models. Those eQTLs that were determined to be significant (FDR adjusted p-value < 0.01) by both models had genetic effect size estimates and corresponding standard errors that largely agreed without any direct bias (Fig 6A and 6D). For the eQTLs that were significant in the robust model (Fig 6B and 6E), but not in the conventional model, the standard error estimates appeared to be inflated in the conventional linear model. This is an indication that at least for a subset of the models the error term might not be Gaussian, but instead more heavy-tailed or potentially contaminated by extreme values or outliers. In the case of eQTLs that were found to be significant only in the conventional model (Fig 6C and 6F), we observed an inflation of the genetic effect size estimates, ($|\hat{\beta }|$), which is likely due to one or a subset of observations. Visualisation of the standard error estimates suggests that estimates are larger for the robust model, however, this is likely directly linked to the biased effect size estimates in the conventional model.

To further investigate potential trends relating to the discordance of the eQTL associations found by the two models, we tested the error term of each eQTL model for non-Normality. We found that the proportion of eQTL models where normality of the error term were rejected by the Anderson-Darling test (at 5% type I error) was slightly higher in the set of eQTLs that were significant in the robust model only (about 70% compared to about 60% of models that were either significant in both the robust and conventional models, or the conventional model only). This further indicates the non-Normal error term distribution is likely to contribute to reduce power of the conventional model.

### Real biological eQTL data set 2: MuTHER

**Fig 7:**
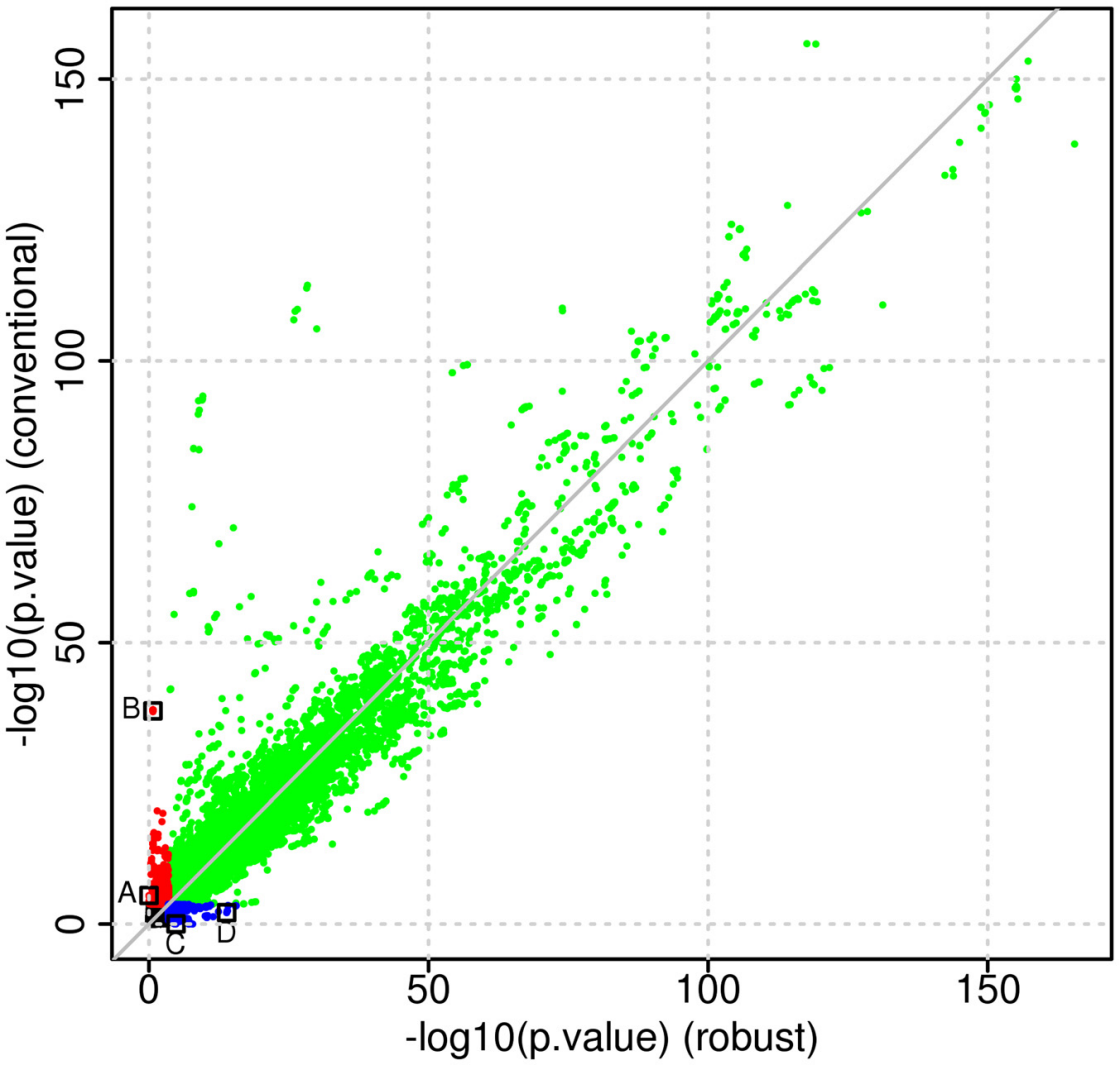
P-value correspondence in Grundberg *et al*. data set [26]. Scatter plot of −*log*_10_(p-values) from MuTHER data set [26]. (Key: green = significant in both models, red = significant in the conventional model only, blue = significant in the robust model only, data from points marked with black squares are shown in S1 Fig).

The second biological eQTL data set was from the MuTHER study and includes adipose tissue data from 449 unrelated individuals which were analysed here, see [26] for details. We found that the concordance between the conventional and the robust models were relatively high (91%), see Table 2, a substantially higher concordance than in the Myers *et al*. data set (50%). A scatter plot of the p-values from the conventional and robust model can be found in Fig 7. The results indicated a relatively high degree of concordance between the two models and particularly for the smallest p-values, although there are also numerous discordant p-values. Examples of discordant eQTL models (labelled in Fig 7) can be found in (S1 Fig). The concordance of genetic effect size estimates and associated standard errors (S2 Fig) between the two models show similar patterns as for the previous data set (Myers *et al*.).

**Table 2:** Concordance (number and proportion of mRNAs with at least one eQTL SNP) between the conventional and robust models (MuTHER data set [26]).

	Both	L.M. unique	Rob. unique
Proportion eQTLs	0.91	0.03	0.06
Number eQTLs	1272	48	83

Key:both = significant in both models, L.M. unique = significant in conventional model only, Rob. unique = significant in robust model only.

## Discussion

EQTL analysis enables detection and characterisation of how genetic variability influence the mRNA expression of individual genes, and has proven an important approach for understanding the genetics of gene expression. Gene expression data are however noisy, both due to the stochastic nature of biological systems and due to technical noise. This inherit noise may invalidate the common assumption of Gaussianity of error terms in, e.g. linear models, which are commonly used in eQTL analysis. If the error term is more heavy-tailed or come from a contaminated Gaussian distribution, this can lead to inflation of both type I and type II errors in the analysis.

Alternatives to the conventional linear additive eQTL model include rank based statistics, e.g. Spearman rank correlation, which provides robustness in many respects. However, rank based statistics have an inherit limitation in that it is not straightforward to adjust the model for relevant covariates. This is a major drawback in many eQTL studies, particularly in human studies, which will have to be adjusted for general covariates representing major phenotypes of the subjects, including gender, age, body mass index and batching effects. Hence, a direct advantage of the robust linear model is that it provides straight forward means for covariate adjustment. Other alternatives to utilising robust models in situations where data are unlikely to comply with assumption of e.g. Gaussianity is to perform careful model checking of each model. It is also important that data are checked carefully and that “multivariate outliers” are removed prior to statistical analysis is carried out in general. We define multivariate outliers as observations designated as outliers based on the complete set of variables measured, e.g. the full set of mRNA measurements across the genome in the case of transcriptomic data and eQTL analysis. Such multivariate outliers can easily be detected by, for example, Principal Component Analysis (PCA), and generally arise due to severe and idiosyncratic technical variability. However, after multivariate outliers have been excluded, which are usually few, there may still be outliers and extreme values present in individual mRNA variables, and these extreme values are often independent between variables. For these “univariate” outliers it is not reasonable to exclude the full observation from all further analysis, since if whole observations were removed due to an extreme value in a single variable little data would be left for analysis. The common approach to ensure that individual models and data are well behaved is through model checking. While manual model checking of a few hundred, maybe up to a thousand models, might be possible, it is unlikely that manual checking of all models in the case of eQTL analysis is tractable. We also note that common model checking procedures based on diagnostic plots might be subjective, which in general is undesirable. Data transformations provide another alternative to reduce the impact of atypical or outlying observations. Example of such transformations includes the Box-Cox transformation and the inverse normal transformation. Transformation of data is, however, not guaranteed to remedy problems originating from noisy data, and may in some circumstances also introduce secondary problems [27]. Another potential drawback of data transformations is that they generally operate on variables marginally, and may therefore not eliminate atypical or outlying observations in the SNP-conditional gene expression distribution. We conclude that there are several approaches to handle noisy data, of which robust methods provide one approach, while alternative methods may provide individual advantages and disadvantages. It is also important to realise that under ideal conditions (i.e. a Gaussian error term) the robust model will have slightly reduced statistical power compared to the conventional model, while under e.g. non-Gaussian conditions, the power of a robust model may be substantially higher. Computing time of the robust linear model is another aspect that has to be considered, particularly in applications such as eQTL analyses. We found the computing time of the robust model to be ∼ 43 times higher compared to the conventional linear model based on the R implementation of the model we applied in this study. The computing time was found to be 0.10s for a single robust eQTL model (N = 500), compared to 0.0023s for the linear model using an Intel Xeon E5-2697 2.70GHz CPU. A typical genome-wide cis-eQTL analysis using the robust model thus requires approximately 1100 cpu hours, assuming 20000 genes and 2000 cis-SNPs/gene on average. On a computing workstation with 16 CPU cores this would correspond to approximately 70-hours run-time, which could be significantly reduced if a computing cluster is used. Thus it is clear that application of robust linear models comes at a higher computational cost. However, the computational time is far from prohibitive, particularly putting the computing time and cost in relation to both experimental time and costs required to generate the molecular data.

From our simulation study we found that even moderate deviations from the model assumptions made in the conventional linear model, e.g. Gaussianity of the error term, can lead to both increase in type I and type II errors. From analyses of two real eQTL data sets we found that residuals in eQTL analysis is not necessarily Gaussian, and that a substantial proportion of the “significant” models found are not in agreement between the conventional linear model and the robust alternative. The results indicate that some type II errors (false negatives) are due to inflated estimates of standard errors due to non-Gaussian error terms. Disagreement in effect size estimates between the two models can lead to both type I errors (false positives) and type II errors (false negatives), and is likely due to extreme values or moderate outliers, as can be seen in examples shown. In the larger of the two eQTL studies (MuTHER), there is a relatively high degree of concordance between the models, which is encouraging. This can probably be explained by a larger sample size, which is expected to reduce the influence of extreme observations and outliers, while the higher concordance might also be explained by difference in quality and homogeneity of the biopsies between the two studies.

In large-scale analyses, such as in the case of genome-wide eQTL analysis, robust statistical models have the potential to provide more reliable results and under some conditions also increased power, for example, in situations with noisy and non-Gaussian data. In situations where data fulfill assumptions of Gaussianity the loss in power by the robust model is relatively minor. Our results suggest that it is likely that model assumptions are violated and that the error term is non-Gaussian in the case of eQTL analysis, and most likely in analyses of many other types of “omics” data as well. We therefore suggest that robust models should be considered as a standard tool for genome-wide eQTL analysis alongside the currently utilised methodologies.

## Supporting Information

S1 Text Example code for fitting robust linear models in R. (R)

S1 Fig Four examples where the conventional and robust models lead to discordant conclusions regarding significance in the MuTHER data set. Labels A-C correspond to marked points in Fig 7. Note that a small amount of random variability have been added in the x-axis direction to better visualise data, in addition to variability originating from the imputation process. MRNA expression levels (y-axis) represent $y_{i}-x_{i}{\hat{\beta }}_{covariates}$, where ${\hat{\beta }}_{covariates}$ are the estimated coefficients for all predictors in the model excluding the genetic effect, i.e. the mRNA signal after adjusting for covariates. Since the conventional and robust estimates of ${\hat{\beta }}_{covariates}$ will be different, the data points in the plots for the robust and conventional models will not be identical.
(TIFF)

S2 Fig Concordance of genetic effect size estimates and associated standard errors between the conventional and robust models in the MuTHER data set. SNP effect size estimates and standard errors for eQTLs significant in both models (A, D), in the robust model only (B, E), and in the linear model only (C, F).
(TIFF)

S1 Table Type-I error rates under the contamination model. (PDF)

S2 Table Estimates of *β* under contamination model. (PDF)

S3 Table Type-I error rate under the heavy-tailed model. (PDF)
