# Supplementary figures and images for: Robust Linear Models for Cis-eQTL Analysis
Source: PLoS One. 2015 May 18;10(5):e0127882. doi: 10.1371/journal.pone.0127882 (PMC4436354; doi:10.1371/journal.pone.0127882)

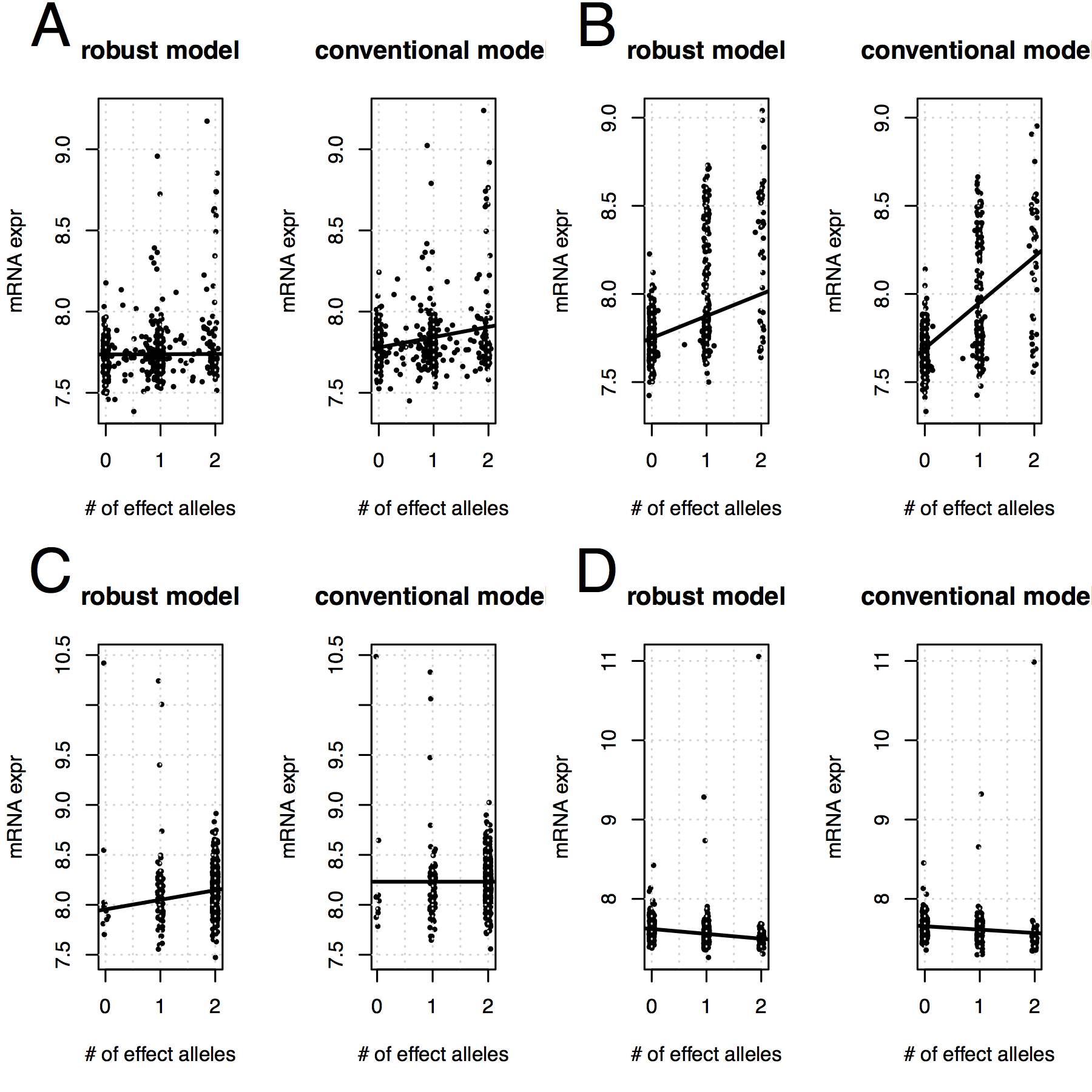

Supplement: S1 Fig — Labels A-C correspond to marked points in Fig 7. Note that a small amount of random variability have been added in the x-axis direction to better visualise data, in addition to variability originating from the imputation process. MRNA expression levels (y-axis) represent yi−xiβ^covariates, where β^covariates are the estimated coefficients for all predictors in the model excluding the genetic effect, i.e. the mRNA signal after adjusting for covariates. Since the conventional and robust estimates of β^covariates will be different, the data points in the plots for the robust and conventional models will not be identical. (TIFF) [file pone.0127882.s002.tiff]

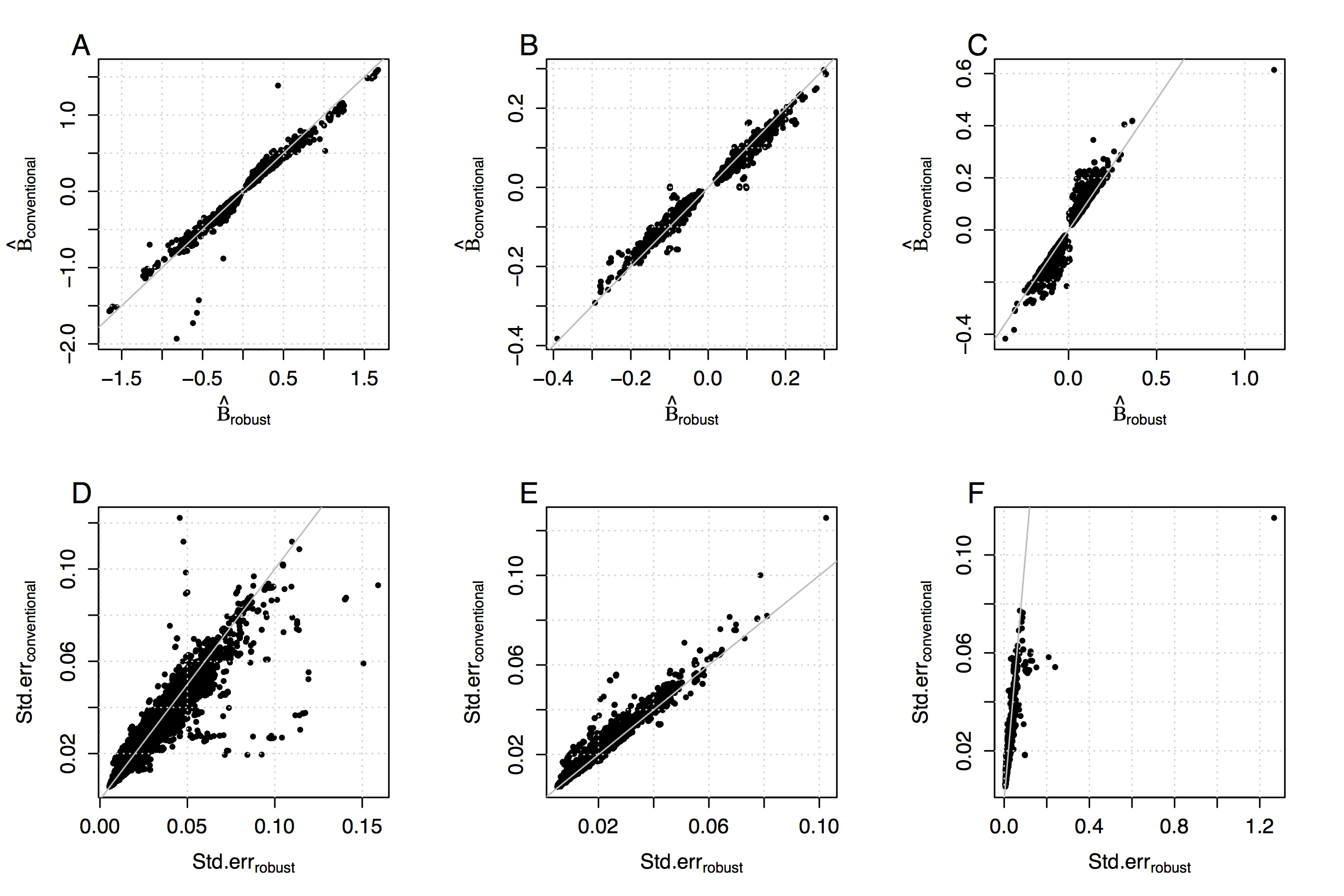

Supplement: S2 Fig — SNP effect size estimates and standard errors for eQTLs significant in both models (A, D), in the robust model only (B, E), and in the linear model only (C, F). (TIFF) [file pone.0127882.s003.tiff]
