# Supplementary material for: Robust Linear Models for Cis-eQTL Analysis
Source: PLoS One. 2015 May 18;10(5):e0127882. doi: 10.1371/journal.pone.0127882 (PMC4436354; doi:10.1371/journal.pone.0127882)
Supplement: S1 Table — (PDF) [file pone.0127882.s004.pdf]

Supplementary Table 1: Simulation study 1 - contamination model

alpha	1-contamination	lm(cont)	rob.lm(cont)	lm(no cont)	rob.lm(no cont)
0.050	0.90	0.049658	0.050639	0.049943	0.050303
0.050	0.92	0.049671	0.050490	0.050051	0.050398
0.050	0.94	0.049436	0.050168	0.050148	0.050395
0.050	0.96	0.049234	0.050143	0.049997	0.050110
0.050	0.98	0.050154	0.050386	0.050245	0.050305
0.050	1.00	0.050226	0.050499	0.050226	0.050499
0.010	0.90	0.009504	0.010202	0.009993	0.010241
0.010	0.92	0.009540	0.010387	0.010160	0.010340
0.010	0.94	0.009542	0.010312	0.010138	0.010379
0.010	0.96	0.009485	0.010233	0.010026	0.010219
0.010	0.98	0.009879	0.010375	0.010056	0.010259
0.010	1.00	0.010131	0.010403	0.010131	0.010403
0.001	0.90	0.000862	0.001032	0.000991	0.001070
0.001	0.92	0.000892	0.001093	0.001022	0.001112
0.001	0.94	0.000865	0.001084	0.001017	0.001082
0.001	0.96	0.000911	0.001087	0.001027	0.001104
0.001	0.98	0.000964	0.001068	0.001028	0.001087
0.001	1.00	0.001038	0.001122	0.001038	0.001122

Type-I error rates under the contamination model. Key: alpha = significance level, contamination = 1-contamination proportion, lm(cont) = type I error rate for the conventional model and contaminated data, rob.lm(cont) = type I error rate for the robust model and contaminated data, lm(no cont) = type I error rate for the conventional model and Gaussian data, rob.lm(no cont) = type I error rate for the robust model and Gaussian data.
