# Supplementary material for: Robust Linear Models for Cis-eQTL Analysis
Source: PLoS One. 2015 May 18;10(5):e0127882. doi: 10.1371/journal.pone.0127882 (PMC4436354; doi:10.1371/journal.pone.0127882)
Supplement: S2 Table — (PDF) [file pone.0127882.s005.pdf]

Supplmenetary Table 2: Simulation study 1 - contamination model

	mean	sd
conventional (cont.)	0.4989	0.1702
robust (cont.)	0.4986	0.1528
conventional (no cont.)	0.4996	0.1469
robust (no cont.)	0.4993	0.1505

Estimates of β under the contamination model
