# Supplementary material for: Robust Linear Models for Cis-eQTL Analysis
Source: PLoS One. 2015 May 18;10(5):e0127882. doi: 10.1371/journal.pone.0127882 (PMC4436354; doi:10.1371/journal.pone.0127882)
Supplement: S3 Table — (PDF) [file pone.0127882.s006.pdf]

Supplementary Table 3: Simulation study 2 - heavy-tailed model

alpha	DF	lm(cont)	rob.lm(cont)	lm(no cont)	rob.lm(no cont)
0.050	2	0.046566	0.050815	0.049803	0.049894
0.050	4	0.049715	0.050350	0.049955	0.050171
0.050	6	0.049937	0.049986	0.050156	0.050273
0.050	8	0.049929	0.050165	0.049830	0.049974
0.050	10	0.049776	0.049945	0.049995	0.050010
0.050	25	0.049862	0.049844	0.050154	0.050317
0.010	2	0.007710	0.010209	0.009974	0.010226
0.010	4	0.009452	0.010142	0.010013	0.010195
0.010	6	0.009785	0.010153	0.010006	0.010200
0.010	8	0.009946	0.010228	0.009968	0.010195
0.010	10	0.009921	0.010240	0.009958	0.010263
0.010	25	0.009904	0.010163	0.009907	0.010194
0.001	2	0.000556	0.001034	0.001021	0.001082
0.001	4	0.000852	0.001013	0.001035	0.001073
0.001	6	0.000949	0.001039	0.000975	0.001067
0.001	8	0.000978	0.001064	0.001019	0.001101
0.001	10	0.000961	0.001043	0.000941	0.001051
0.001	25	0.001008	0.001098	0.000967	0.001046

Type-I error rate heavy-tailed model. Key: alpha = significance level, DF = degrees of freedom of the error term t-distribution, lm(cont) = type I error rate for the conventional model and contaminated data, rob.lm(cont) = type I error rate for the robust model and contaminated data, lm(no cont) = type I error rate for the conventional model and Gaussian data, rob.lm(no cont) = type I error rate for the robust model and Gaussian data.
